# Cough in hypereosinophilic syndrome: case report and literature review

**DOI:** 10.1186/s12890-020-1134-x

**Published:** 2020-04-15

**Authors:** Jiaxing Xie, Jianheng Zhang, Xiaoxian Zhang, Qingling Zhang, Kian Fan Chung, Chunyan Wang, Kefang Lai

**Affiliations:** 1 Department of Allergy and Clinical Immunology, National Clinical Research Center for Respiratory Disease, State Key Laboratory of Respiratory Diseases, Guangzhou Institute of Respiratory Health, The First Affiliated Hospital of Guangzhou Medical University, Guangzhou, China; 2 National Heart and Lung Institute, Imperial College London & Royal Brompton Biomedical Research Unit at Royal Brompton and Harefield NHS Foundation Trust, London, UK; 3 Department of hematology, The First Affiliated Hospital of Guangzhou Medical University, 1 Kangda Road, Guangzhou, 510230 China; 4 National Clinical Research Center for Respiratory Disease, State Key Laboratory of Respiratory Disease, Guangzhou Institute of Respiratory Health, the First Affiliated Hospital of Guangzhou Medical University, 151 Yanjiang Road, Guangzhou, 510120 China

**Keywords:** Airway eosinophilic inflammation, Cough, Hypereosinophilic syndrome, Imatinib, *PDGFRA* fusion gene

## Abstract

**Background:**

Cough and airway eosinophilic inflammation has not been highlighted in hypereosinophilic syndrome (HES).

**Case presentation:**

We report 2 further cases and reviewed the clinical features and treatment of HES present with cough from the literature. Both cases were middle age male, presenting with chronic cough, airway eosinophilic inflammation and hyper eosinophilia who have been previous misdiagnosed as cough-variant asthma and failed anti-asthma treatment. *PDGFRA* fusion gene was confirmed in one case, but not in the other case. Both had evidence of myeloproliferative features. The tyrosine kinase inhibitor, imatinib, resulted in complete resolution of eosinophilia and cough. By searching PubMed, we found 8 HES cohorts of 411 cases between 1975 and 2013, where the incidence of cough was 23.11%. Sixteen case reports of HES presented with cough as predominant or sole symptom, with nine male patients with positive *PDGFRA* fusion gene, who responded well to imatinib. Six of seven patients, who tested negative for the *PDGFRA*, responded to systemic glucocorticoids.

**Conclusions:**

Cough and airway eosinophilic inflammation is common in some HES patients. *PDGFRA*+ HES patients present with chronic cough respond well to imatinib. Our case reports indicate that *PDGFRA* negative HES patients may respond to imatinib as well.

## Background

Chronic cough is defined as the sole or predominant symptom and lasting for more than 8 weeks, with a normal chest x-ray [1]. The common causes of chronic cough are cough-variant asthma (CVA), non-asthmatic eosinophilic bronchitis (NAEB), upper airway cough syndrome and gastroesophageal reflux disease (GERD) [2]. Eosinophilic airway inflammation is commonly observed in chronic cough, usually responding to corticosteroids [3].

Recently, two outstanding reviews [4, 5] describe the emerging role of eosinophilic inflammation in chronic cough, new insights on its mechanisms and available treatments. These two reviews basically focused on eosinophilic airway inflammation in asthma, nonasthmatic eosinophilic bronchitis, and upper airway cough syndrome. However, the hypereosinophilic syndrome (HES) could be a rare and long-ignored cause of chronic cough. HES comprises a heterogeneous group of hematologic disorders characterized by unexplained sustained eosinophilia (> 1500/μL for more than 6 months) associated with signs and symptoms of organ involvement [6]. While HES is a rare disease, HES presenting with chronic cough as the main symptom is even rarer. HES patients may have eosinophilic infiltrates in the airways, and are often misdiagnosed as CVA, asthma, or other eosinophilic lung diseases. Chronic cough as presenting manifestation of *platelet-derived growth factor receptor alpha* (*PDGFRA*) + chronic eosinophilic leukaemia is being increasingly recognized [7]. Recently, we successfully treated 2 patients with HES, eosinophilic airway inflammation, and chronic cough, in whom one was *PDGFRA* +, but the other was not. We report both cases and reviewed all published cases and cohorts in literature to learn more about the features of HES-associated cough.

## Case presentation

### Case 1

**Fig. 1:**
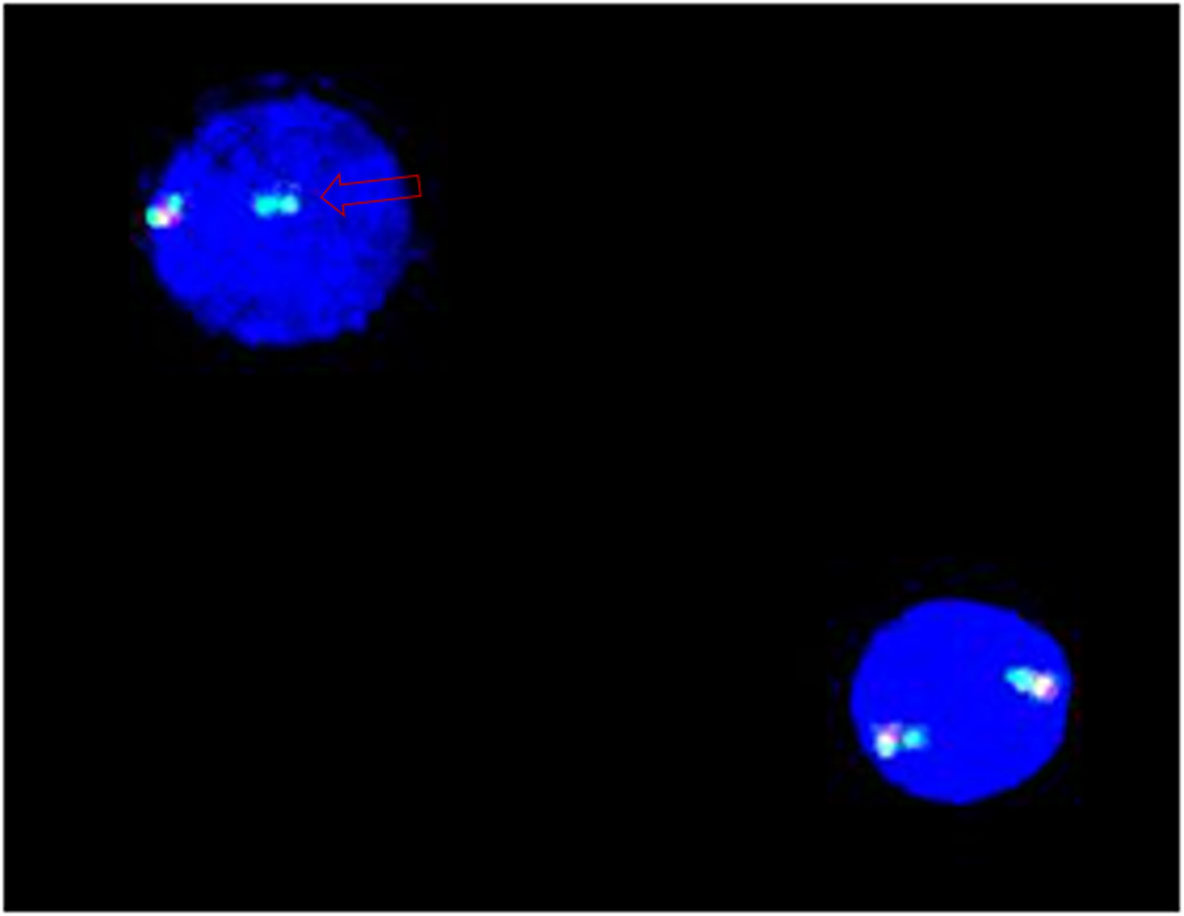
Fluorescence in situ hybridisation (FISH) analysis of case 1. Absence of the *CHIC2* region was observed as loss of a red signal (arrow) from the co-localized green/green signal, indicative of the presence of this specific deletion that leads to *FIP1L1-PDGFRA* fusion on one of the chromosomes 4

The patient was a 41-year-old male with 20 pack-years smoking history who complained of a chronic cough that had lasted for more than 2 years with shortness of breath, for 6 months. His cough was worse at night and was aggravated in the supine position. Auscultation of the lung was normal. There was a grade 3 systolic murmur at the apex and in the area of the tricuspid valve and mild pitting edema was seen in both lower limbs. The blood eosinophil count was 7510/μL. The cardiac shadow was enlarged, and there was a small pericardial effusion in chest computed tomography (CT). Forced expiratory volume in the first second (FEV_1_) was 97.63% of predicted value, with FEV_1_/FVC was 100.97%. Peak expiratory flow variability over 1 week was 27%. Bronchoscopy was normal, but bronchoalveolar lavage fluid (BALF) indicated 28% eosinophils. Total IgE was 26.1 kU/L. CVA was initially suspected by another respirologist who performed initial diagnostic workups including bronchoscopy according to the presence of airway reversibility and airway eosinophilia. Methylprednisolone at 80 mg/d IV and bronchodilators were given. But the symptoms did not improve, and the eosinophil count remained elevated at 10,700/μL. He was referred to our hospital. The B-type natriuretic peptide (BNP) was 4776 pg/mL, and the antineutrophil cytoplasmic antibody was negative. Cardiac magnetic resonance imaging showed hypertrophic cardiomyopathy. Coronary angiography showed no significant stenosis in the coronary arteries. Abdominal ultrasound showed an abdominal effusion and splenomegaly. The patient was treated with inhaled corticosteroids (ICS), cardiotonic drugs, and diuretics, leading to a slight improvement in symptoms. Antibody for both paragonimiasis and liver flukes were positive. Praziquantel was given, without improvement. The bone marrow cytology showed eosinophilia (37.5%). The test for the *PDGFRA* fusion gene mutation was positive (Fig. 1). Imatinib tablets 100 mg daily were given. Because the patient had cardiac involvement and elevated BNP level, dexamethasone 10 mg daily was administered at the same time. The dose of corticosteroid was gradually tapered off. The patient’s dry cough and shortness of breath were relieved. The blood eosinophil dropped to 60/μL. An echocardiogram was repeated 4 months after discharge and showed no improvement. Mitral tricuspid valve angioplasty was performed, with improvement of his cardiac function. The final diagnosis was myeloid and lymphoid neoplasm associated with eosinophilia and *PDGFRA* rearrangement.

### Case 2

**Fig. 2:**
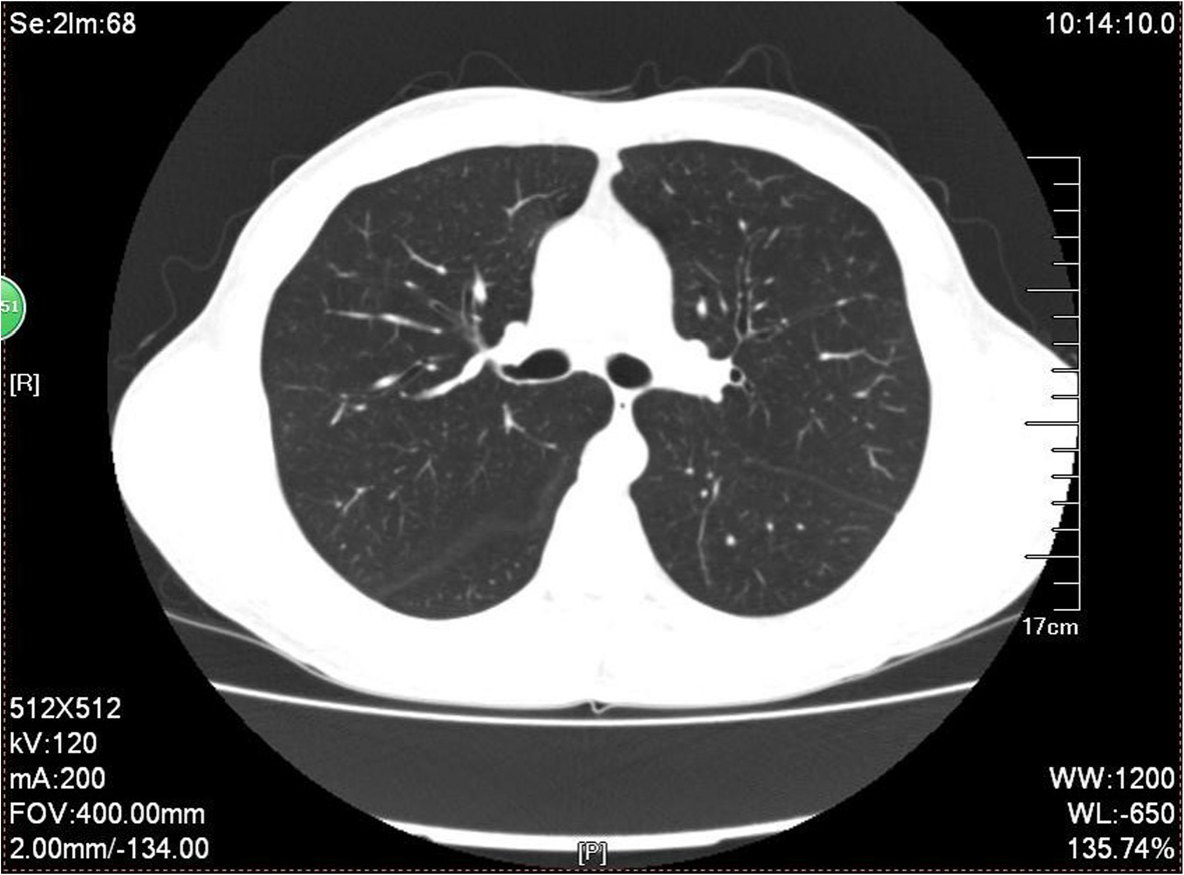
The chest CT of case 2. The chest CT showed multiple scattered nodules in both lungs

The patient was a nonsmoker 52-year-old male with a chronic productive cough for 7 years after moving into a new office. The cough was more pronounced during the night and worsened on exposure to cigarette smoke. The sputum was white and sticky and not easy to cough up. At that time blood eosinophils count was 2220/uL(36.2%). FEV_1_ was 98.3% of predicted value. FEV_1_/FVC was 70.04%. The methacholine (MCh) challenge test was positive (PD_20_ = 2.504 mg). The induced sputum eosinophil count was 64%. The patient was diagnosed as CVA and treated with inhaled budesonide formoterol, but his symptoms did not improve. He was then given oral montelukast sodium and prednisone 10 mg daily and his symptoms improved slightly. However, his eosinophil count did not decrease. He was then admitted to the hematology department. The bone marrow biopsy showed eosinophilia. Prednisone 40 mg daily was given, and the cough was slightly improved. The patient continued to take prednisone intermittently for 4 years. However, his eosinophil count was still up to 4020/μL. The patient was admitted to the respiratory department of our hospital due to cough and hyper eosinophilia. Dry rales were heard on exhalation. The blood eosinophil was 1550/uL(27.0%). No abnormalities were found on a CT of the paranasal sinuses. The chest CT showed multiple scattered nodules in both lungs (Fig. 2). The T total IgE was 157 kU/L. BNP and troponin were normal. The induced sputum eosinophil count was 18.5%. Fractional exhaled nitric oxide (FeNO) was 96 ppb. FEV_1_ was 110% of predicted value. FEV_1_/FVC was 70.84%. The MCh bronchial challenge test was positive (PD_20_ = 1.877 mg). Biopsy of the bronchial mucosa and gastric mucosa showed eosinophilic infiltration. The ultrasound indicated splenomegaly (126 mm long). Parasite antibodies were negative. Since the patient had a history of eating raw river fish, he was given albendazole 0.4 daily for 1 week and inhaled beclomethasone/formoterol. There was no significant remission of cough and no decrease in the eosinophil count. Finally, he was transferred to the hematology department. An ultrasound indicated that the spleen (142 mm long) was larger than previously. The bone marrow aspiration showed eosinophilia. The eosinophil percentage in the peripheral blood was 28%, and precursors were also seen. Examination for *PDGFRa, PDGFRb, FGFR, JAK2* or *FLT3* fusion genes proved negative. Imatinib 0.1 g per week was given, and the cough subsided significantly 1 month after this treatment. Two months later blood eosinophil was 120/uL(2%). At the final follow up the cough was completely relieved for the first time in 7 years. This patient was diagnosed as *PDGFRA* negative HES associated myeloid neoplasm (MHES).

### Literature search strategy and terms

For the literature review, we used the PubMed database. Papers written in English were selected. Main search terms were *hypereosinophilic syndrome* and *cough*. Additional papers were included by reviewing the references of the primarily selected papers with the same criteria. Cases with recognized pediatric HES, specific disease entities including Churg Strauss vasculitis, acute or chronic eosinophilic pneumonia, allergic bronchopulmonary aspergillosis, and other secondary hypereosinophilia were excluded.

Eight cohorts of HES patients consisting of 411 cases were retrieved from PubMed [8–15] (Table 1). The lung involvement ranged from 25 to 67% (37.77%), with an incidence of cough ranging from 10 to 41% (23.11%). In 2 of the 8 studies, 69 patients, all male, were positive for the *PDGFRA* fusion gene [8, 9] with the incidence of cough being 37.68%. In 2 other studies, positivity for the *PDGFRA* fusion gene was lower (about 10%) [10, 11], with ratio of male to female being similar, and the incidence of cough was 20.67%. Four studies were reported in the 1970s and 1980s [12–15], at that time no method for the detection of the *PDGFRA* fusion gene was available. The cough incidence in these 4 studies was 29.52%. In the aforementioned cohorts, no measurement of airway eosinophilia was performed to assess the severity of eosinophilic inflammation.

**Table 1:** Frequency of pulmonary involvement and cough in HES from 6 series

Author	Year of publication	Cases	Sex (M/F)	Age (y)	Pulmonary involvement (%)	Cough (%)	*PDGFRA* fusion gene positive (%)
Helbig [5]	2013	25	23/2	NA	NA	8/25 (32)	25/25 (100)
Legrand [6]	2013	44	43/1	41 (6–67) ^a^	20/44 (45)	18/44 (41)	44/44 (100)
Dulohery [7]	2011	49	25/24	50 (12–88) ^a^	33/49 (67)	19/49 (39)	4/49 (8)
Ogbogu [8]	2009	188	104/84	45 (6–85) ^a^	47/188 (25)	19/188 (10)	18/161 (11)
Spry [11]	1983	15	13/2	32.2	12/15 (40)	12/15 (40)	NA
Fauci [9]	1982	50	NA	33^b^	20/50 (40)	12/50 (24)	NA
Parrilo [12]	1979	26	NA	NA	NA	3/26 (12)	NA
Chusid [10]	1975	14	14/0	37.5^b^	6/14 (43)	4/14 (29)	NA

*NA* not available

^a^Median (range); ^b^ mean

We found 16 HES cases [7, 16–30] with cough as the main or only symptom (Case 3–18, Table 2). The overall clinical information is summarized in Table 2. The average age was 53.6 years, and the male-to-female ratio was 13:3. There were 2 current smokers, 1 ex-smoker, 5 nonsmokers, and 6 unknown status. All patients had elevated blood eosinophil counts, with an average of 7800/uL. The eosinophils in BALF were significantly elevated when evaluated, ranging from 20 to 84%.

**Table 2:** Clinical summary of the 18 cases of hypereosinophilic syndrome presenting as cough

Case	Age(y)/sex	Smoking	Symptoms/duration of cough	Eos count in blood (/μL)	TIgE kU/l	BALF Eos%	Induced sputum Eos%	Lung function results	Chest imaging	Cardiac dysfunction	Result of *PDGFRA* fusion gene	Treatment
1^a^	41 M	20 pack-years	Chronic dry cough/2 y	7510	26.1	27	NA	FEV_1_, FEV_1_%: nl	CXR: normal lung field, CT: nl	Yes	Pos	Imatinib
2^a^	52 M	Nonsmoker	Chronic cough/7 y	2220	157	NA	64 at the beginning 18.5 7 years later	Mild obstructive, bronchial responsiveness. At the beginning and 7 years later	CXR: nl. 7 years later, CXR: nl, CT: multiple scattered nodules	No	Neg	Imatinib
3 [16]	42 M	NA	Chronic dry cough/1 y	3560	65.7	NA	^b^	FEV_1_, FEV_1_% and DLCO: nl	CXR: nl	No	Pos	Imatinib
4 [17]	54 M	stopped smoking, 15 y	Chronic cough/2 y	5000	61	NA	NA	FEV_1_, histamine bronchial responsiveness and DLco: nl	CT: thickening of intrapulmonary airways with distal airway plugging	No	Pos	Imatinib
5 [7]	65 M	Active smoker	Chronic incapacitating cough/4.5 y	5180	19	20	NA	No airway obstruction, decreased DLCO, marked airway hyperreactivity	CT: nl	No	Pos	Imatinib
6 [18]	55 M	Never smoker	Non-productive cough/ 7 mo	12,700	nl	NA	NA	Spirometry and transfer: nl	CXR: nl; chest CT: patchy bronchocentric consolidation	No	Pos	Imatinib
7 [19]	57 M	NA	Chronic dry cough/2.5 y	4680	NA	NA	NA	nl	CXR: nl; chest CT: nl	No	Pos	Imatinib
8 [20]	46 M	NA	Persistent dry cough, progressive dyspnea	12,300	NA	NA	NA	NA	CXR: cardiac enlargement and bilateral pleural effusion	Yes	Pos	Imatinib Methylprednisolone
9 [21]	32 M	Nonsmoker	Shortness of breath, cough/4 mo	12,500	NA	NA	NA	Mild restrictive and severe obstructive lung disease	CT: tree-in-bud and ground-glass opacities	No	Pos	Imatinib
10 [22]	45 M	NA	Dyspnea, cough	8020	NA	NA	NA	NA	CXR: bilateral pulmonary infiltrates with minimal pleural effusion CTPA: pulmonary embolism	Yes	Pos	Imatinib Methylprednisolone
11 [29]	47 M	NA	Dyspnea, cough	NA	NA	NA	NA	Abnormal diffusion capacity and lung volumes	CT: interstitial infiltrates	NA	Pos	Imatinib
12 [30]	45 M	NA	Chronic cough	NA	NA	NA	NA	NA	NA	NA	NA	Inhaled bronchodilator Imatinib
13 [23]	87 F	Never smoker	Long-term cough	8200	nl	73	NA	Unreliable	CXR: bibasal alveolar infiltrate	No	NA	Methylprednisolone
14 [24]	50 F	Smoking	Recurring dry cough, chest tightness, wheezing	4000	948^⁂^	NA	NA	Mild obstructive and moderate restrictive pattern	CXR: bilateral hilar enlargement	Yes	Neg	Deflazocort
15 [25]	33 M	NA	Progressive dyspnea, cough	15,100	NA	84	NA	NA	CXR: bilateral pulmonary infiltrates	Yes	Neg	Prednisone
16 [28]	42 M	NA	Nonproductive cough/2 mo	8000	NA	NA	NA	NA	CXR: right lower lobe infiltrate	Yes	Neg	Mepolizumab
17 [26]	88 F	Nonsmoking	Persistent nonproductive cough, shortness of breath	7980	141	NA	NA	NA	CXR: nl. 7 mo later, CXR: bilateral patchy infiltrates, CT: bilateral lung infiltrates	Yes	NA	Prednisone
18 [27]	68 M	Former smoker	Recurrent cough, wheezing, shortness of breath/14 mo	3000	2897	NA	NA	nl	CT: slightly enlarged mediastinal, right hilar and bilateral axillary lymph nodes	No	NA	Prednisone

*BALF* bronchoalveolar lavage fluid, *CT* computed tomography, *CTPA* CT pulmonary angiogram, *CXR* chest x-ray, *DLCO* diffusing capacity of the lungs for carbon monoxide, *Eos* eosinophilia, *FEV*_*1*_ forced expiratory volume in 1 s, *FVC* Forced vital capacity, *nl* normal, *NA* not available

^a^The present cases

^b^ with numerous eosinophils in sputum

Nine of twelve cases evaluated were found to have *PDGFRA* fusion gene (case 3–11). There were 10 HES cases with *PDGFRA* fusion gene include our cases (case 1, Table 2), all of them had chronic cough as the main or sole manifestation. They were all male with an average age of 47.7 years. In five cases where total IgE was measured, this was not high. All except case 9 and case11 had normal spirometry. Only two cases (case 4 and 5) performed airway responsiveness test, case 5 had marked bronchial hyperreactivity. Six cases had chest X ray, and 4 cases had normal chest X-ray. Only case 1 and 3 had performed induced sputum test and had significant sputum eosinophils. Another two cases had significantly increased eosinophils in BALF (27% in case 1, 20% in case 5). On airway mucosal biopsy, 2 cases showed eosinophil infiltration in the airway mucosa (case 3 and 4). All cases had been transferred to a number of hospitals and had been alternatively diagnosed with asthma, GERD, pneumonia, and other diseases. Five of them received oral corticosteroids (OCS) (case 1,3, 4, 6, and 9). Three received ICS (cases 1,3 and 5). Two received proton pump inhibitors (cases 4 and 5), and two cases received experimental antiparasitic treatment (cases 1 and 4). All these treatments were ineffective. Eventually, imatinib was given and proved to be effective. Case Two in our study had severe and persistent airway eosinophil inflammation, persistent bronchial hyperreactivity, negative fusion genes and was steroid (OCS and ICS) refractory, but he showed features suggestive of a myeloproliferative neoplasm (progressive splenomegaly and presence of early myeloid precursors on the peripheral smear). We reviewed the clinical feature of case 12 in Table 2 and found it consistent with MHES too. The cough of this case resolved within 3 days of imatinib, and his diffusion capacity, lung volumes and interstitial infiltrates in CT normalized after 3 months of imatinib therapy.

In other six patients [23–28, 30], three showed negative *PDGFRA* fusion gene (cases 14 to 16), and another 3 patients did not have fusion gene examination. The male-to-female ratio was 4:3, and the mean age was 61.3 years. Almost all cases had abnormal chest X ray. Only two cases had performed BALF and showed significantly increased eosinophils (73% in case 13, 84% in case 15). There were 4 cases with cardiac dysfunction. The main treatment was systemic glucocorticoids. After treatment, cough and other symptoms were relieved. However, 4 cases (case 13, 15, 17, and 18) relapsed after the corticosteroid was tapered off. In case 16, her symptom and eosinophilia persisted despite sequential trials of imatinib and interferon-α, but a trial of mepolizumab was effective.

## Discussion and conclusions

In recent years, with the availability of *PDGFRA* fusion gene examination, more HES patients who test positive for this gene have been identified. These patients have often spent years with a misdiagnosis. It is important to clarify whether there is a rearrangement of the fusion gene by early fluorescence in situ hybridization (FISH) or nested RT-PCR testing [31] after eliminating secondary causes of eosinophilia. Because eosinophil autofluorescence may interfere with FISH, Roufosse et al. [7] recommended that both FISH and RT-PCR should be performed when this disease is suspected. Imatinib was curative in HES patients with a positive *PDGFRA* fusion gene, and there was no relapse report. Cardiac structural abnormalities may not improve with imatinib therapy [32], case one didn’t show improvement in cardiac ultrasound after 4 month imatinib and needed mitral tricuspid valve angioplasty. Currently all HES-associated chronic cough cases have only *PDGFRA* fusion gene rearrangements. The other fusion genes rearrangements, such as *PDGFRB*, *FGFR1*, and *JAK2* have not been reported in HES-associated cough cases.

Recently Khoury and Klion et al. [33, 34] proposed *PDGFRA*-negative HES with features suggestive of a myeloproliferative neoplasm (MHES), these patients have splenomegaly, presence of early myeloid precursors on the peripheral smear, elevated serum B12, and/or Tryptase levels, and resistance to corticosteroid therapy. They performed a prospective study of imatinib in HES, found clinical features of MHES predict imatinib response in *PDGFRA* negative HES [33]. Our Case 2 is consistent with MHES. The effect of imatinib is obvious, confirming the great possibility of MHES.

The HES patients without the *PDGFRA* fusion gene are commonly treated with systemic glucocorticoids, though the recurrence rate is high. The treatment of targeted eosinophil depletion, as with the monoclonal anti-interleukin-5 antibody mepolizumab, can be considered. Although previous studies found that mepolizumab failed to reduce cough in patients with refractory eosinophilic asthma [35], it was effective in case 16 [28]. Mepolizumab may become a treatment option for HES patients without the *PDGFRA* fusion gene.

Because the lung is one of the common involved organ of HES, biomarkers of airway eosinophilic inflammation such as FENO, induced sputum, BALF should be recommended in this condition. In Table 2, biomarkers of airway eosinophilic inflammation were measured only in seven patients, and all had increased eosinophil numbers. The cause of cough in HES patients may be associated with high airway and blood eosinophil counts. The cough may be caused by thickening of the basement membrane, goblet cell hyperplasia, or airway eosinophil inflammation [36]. The mast cells may also be involved in the occurrence of cough and be the target of imatinib. It has been confirmed that numbers of active mast cells appear in the bone marrow of HES patients with a positive *PDGFRA* fusion gene [34]. The mechanisms underlying eosinophilic airway inflammation in chronic cough due to allergic (T helper type 2 cells) and nonallergic (innate lymphoid type 2 cells) pathways had been illustrated in 2 recent outstanding reviews [4, 5]. Activated T helper type 2 or innate lymphoid type 2 cells release interleukins, causing eosinophilia inflammation, and bronchial hyperreactivity. Although *PDGFRA+* HES or MHES (case 1–12, Table 2) could showed bronchial hyperreactivity, they had no response to OCS and ICS. It seems no allergic triggers contribute to the pathogenesis. The mechanism underlying eosinophilic airway inflammation and bronchial hyperreactivity warrants further investigation.

In addition, the heart is also one of the most commonly affected organs in HES patients. Our review of the literature found that 7 patients (46.6%) with normal lung function had cardiac insufficiency. The pulmonary edema caused by cardiac insufficiency might be the cause of cough.

In conclusion, cough is one of the main symptoms in HES patients. Eosinophilic airway inflammation and bronchial hyperreactivity are observed in some HES patients and don’t response to corticosteroids. A screening test for the *PDGFRA* fusion gene is essential for patients with increased eosinophils, especially if they are male. For the *PDGFRA* negative ones, MHES should be evaluated. Our case report indicates that cough and eosinophilic airway inflammation in *PDGFRA* negative HES may respond to imatinib as well. Taken together, we suggest the importance of rapid and correct diagnosis of HES and MHES, particularly when only cough or other nonspecific symptoms are present.

## Data Availability

All data discussed in the manuscript are included within this published article.

## Abbreviations

BALF: Bronchoalveolar lavage fluid
BNP: B-type natriuretic peptide
CT: Computed tomography
CVA: Cough-variant asthma
FENO: Fractional exhaled nitric oxide
FEV_1_: Forced expiratory volume in the first second
FVC: Forced vital capacity
FISH: Fluorescence in situ hybridization
GERD: Gastroesophageal reflux disease
HES: Hypereosinophilic syndrome
ICS: Inhaled corticosteroids
MCh: Methacholine
MHES: *PDGFRA* negative HES associated myeloid neoplasm
NAEB: Non-asthmatic eosinophilic bronchitis
OCS: Oral corticosteroids
